# Public health impact of nirsevimab and reduction of RSV hospitalisation in all infants: early real-world data from Tuscany (Italy) in the 2024–25 RSV season

**DOI:** 10.1007/s00431-025-06588-6

**Published:** 2025-11-03

**Authors:** Francesco Nieddu, Marina Vignoli, Emanuela Ferraro, Silvia Boscia, Valeria Astorino, Caterina Pelosi, Valentina Guarnieri, Francesca Quaranta, Vanessa Perone, Paolo Biasci, Lorenzo Lodi, Silvia Ricci, Carlo Dani, Giuseppe Indolfi, Pierre Bourron, Salvatore Parisi, Chiara Azzari, Maria Moriondo

**Affiliations:** 1 Meyer Children’s Hospital IRCCS, Florence, Italy; 2 Department of Health Sciences, University of Florence, Florence, Italy; 3 Family Pediatrician, FIMP Toscana, Florence, Italy; 4 Neonatal Intensive Care Unit, Division of Neonatology, Careggi University Hospital of Florence, Florence, Italy; 5 Department of Neurosciences, Drug Research and Child Health, University of Florence, Florence, Italy; 6 Department NEUROFARBA, University of Florence, Florence, Italy; 7 Sanofi Vaccines, Lyon, France; 8 Sanofi Vaccines, Milan, Italy

**Keywords:** Hospitalisation, Italy, Nirsevimab, RSV

## Abstract

Real-world evidence on the public health impact of nirsevimab across a full RSV season in Italy is currently lacking, particularly with respect to hospitalisations and admissions to paediatric intensive care units (PICU), and only limited data are available worldwide. This study aimed to evaluate, in a real-world setting, the public health impact of nirsevimab on RSV-related hospitalisations and PICU admissions in Tuscany. This observational retrospective study included all children under one year of age experiencing their first RSV season, i.e., those born between April and March of the 2021–22, 2022–23, 2023–24, and 2024–25 seasons, who were hospitalised at the Meyer Children’s Hospital, a tertiary regional paediatric hospital in Tuscany, for respiratory symptoms with RSV confirmed via real-time PCR testing. Starting November 1st 2024, Nirsevimab was offered to all infants born between 1 April, 2024, and 31 March, 2025. The percentage reduction in RSV-related hospitalisations was calculated by comparing the number of hospitalisations observed in 2024/25 with the mean number recorded across the three preceding RSV seasons. Immunization coverage reached around 90%. During the 2024–2025 RSV season, RSV-related. hospitalisations decreased by 82.1% among infants eligible for immunization, by 83.2% among those born during the RSV season, and by 81.5% among those born before the season. PICU admissions decreased by 85.2%, 84.0%, and 86.8%, respectively, in the same groups. High coverage of nirsevimab immunization substantially reduced RSV-related hospitalisations among infants in Tuscany during the 2024–2025 season. The consistent benefits observed across both in-season and out-of-season birth cohorts support a universal immunization program for all infants, including those born from April onward, in Italy.

**Table:** 

**What is Known:** *• RSV is the leading cause of infant hospitalisations for lower respiratory tract infections, especially in those ≤12 months. Real-world data on season-wide nirsevimab public health impact in Italy were lacking..*
**What is New:** *• Universal nirsevimab immunisation in Tuscany led to an 82.1% reduction in RSV-related hospitalisations and 85.2% in PICU admissions in infants eligible for immunization. Findings support universal over selective immunisation to protect all infants entering their first RSV season.*

**What is Known:**

*• RSV is the leading cause of infant hospitalisations for lower respiratory tract infections, especially in those ≤12 months. Real-world data on season-wide nirsevimab public health impact in Italy were lacking..*

**What is New:**

*• Universal nirsevimab immunisation in Tuscany led to an 82.1% reduction in RSV-related hospitalisations and 85.2% in PICU admissions in infants eligible for immunization. Findings support universal over selective immunisation to protect all infants entering their first RSV season.*

## Introduction

Respiratory syncytial virus (RSV) is a primary cause of lower respiratory tract infections (LRTI) and hospitalisation in infants, particularly under one year of age [1–7]. Nirsevimab (Beyfortus), a monoclonal antibody, was approved by the European Medicines Agency in October 2022 for RSV prophylaxis in infants in their first RSV season and children up to 24 months of age who remain vulnerable to severe RSV disease through their second RSV season [8]. Nirsevimab became available in Italy in November 2024 and the offer was given on a regional basis with a consequent fragmentation of immunisation policy yielding a patchwork, non-homogeneous implementation across regions. Limited data are available [9, 10] but still there is a lack of real-world data.

particularly with regard to hospitalisations and admissions to paediatric intensive care units (PICUs). This study evaluated the public health impact of nirsevimab usage in terms of decreasing RSV-related hospitalisations and PICU admissions during the 2024–25 RSV season at the Meyer Children’s Hospital, a tertiary regional paediatric hospital in Tuscany, reference centre for paediatric infectious disease surveillance. The data were compared with those of the three previous RSV seasons (2021–2024).

## Methods

The study was conducted at Meyer Children’s Hospital, the only tertiary-level paediatric hospital and regional referral centre in Tuscany, which hosts the sole specialised paediatric intensive care unit (PICU) in the region. Other hospitals across Tuscany provide paediatric and neonatal care, and infants may be admitted to these facilities; therefore, this single-centre study does not capture all infants in the region.

The observational retrospective study included all children under one year of age experiencing their first RSV season, namely those born between April and March of the 2021–22, 2022–23, 2023–24, and 2024–25 seasons, who were hospitalised for respiratory symptoms during the RSV season from 1 November 2021, to 31 March 2025, with RSV confirmed via real-time PCR testing [11]. For each season considered, the RSV season was defined from 1 November to 31 March of the following year, and hospitalisations occurring within this period were included in the analysis. For the 2021–22 season, however, the RSV season was considered to start on 1 October 2021 due to an anticipated early onset of the epidemic, according to Lastrucci et al. [12]. Respiratory symptoms were defined as the presence of at least one of the following: cough, nasal congestion/rhinorrhoea, sore throat, sneezing, shortness of breath or increased work of breathing, wheeze, tachypnoea, apnoea, or fever accompanied by ≥ 1 respiratory sign [13].

Starting November 1 st 2024, nirsevimab was offered in Tuscany to all infants born between 1 April, 2024, and 31 March, 2025.

In Tuscany, infants born during the season were immunised in hospital nurseries preferably on the second day of life; the infants born before the season were vaccinated by family paediatricians in early November, akin to standard immunisations.

Coverage was calculated using the electronic immunization registry of the Tuscany region. The same result was obtained by dividing the number of nirsevimab doses administered by the number of births during the same period.

A total of 21,471 infants were born in Tuscany before or during the 2023/2024 RSV epidemic season [12]. The estimated average number of births in Tuscany over the three years 2021–2023 was 22,000 per season, according to ISTAT data [14].

The percentage reduction in RSV-related hospitalisations was calculated by comparing the number of hospitalisations observed in 2024/25 with the mean number recorded across the three preceding RSV seasons.

Considering the seasonality of RSV in Italy, infants were grouped based on birth timing: “born during the season” (born between 1 November and 31 March) and “born before the season” (1 April—31 October). Within the latter, we separately evaluated children born April to July or August to October.

Outcomes reflecting disease severity were also assessed. These included the need for respiratory support, classified according to the highest level required during admission (low-flow oxygen, high-flow nasal cannula, continuous positive airway pressure [CPAP] or helmet ventilation, and endotracheal intubation). Admissions to the paediatric intensive care unit (PICU) were also evaluated. For each outcome, both absolute numbers and incidence rate ratios (IRRs) were calculated, comparing the 2024–25 RSV season with the preceding three seasons (2021–24), separately for infants born during and outside the RSV season.

Statistical analysis was conducted using SPSS version 21.

The reduction in RSV-related hospitalisations and other severity outcomes was assessed using a Poisson exact test, comparing the observed number of cases in the 2024–25 season with the expected number based on the mean of the three preceding seasons. Statistical significance was set at *p* < 0.05.

Moreover, hospitalisation data were available as monthly counts between November and March for each season. We constructed a panel dataset (month × season) covering three pre-intervention seasons (2021–22, 2022–23, 2023–24) and one post-intervention season (2024–25). The primary outcome was the number of RSV-related hospitalisations per month. A negative binomial regression was used, including calendar month as a fixed effect to adjust for intra-seasonal variation and a binary indicator variable for the intervention period (0 = without nirsevimab, 1 = with nirsevimab).

Results are presented as incidence rate ratios (IRR) with 95% confidence intervals (CI).

## Results

A total of 570 infants (247 females) were included in the present study.

### Coverage data

In Tuscany, during the 2024–25 RSV season, the observed coverage of nirsevimab among eligible infants was 87.9%, reflecting the implementation of the regional immunisation programme.

### RSV-related hospitalisations in all infants eligible for immunisation

Among infants eligible for immunisation, RSV-related hospitalisations fell from a baseline mean of 179.3 cases in the three previous seasons (161, 198, 179) to 32 cases in 2024–25, yelding an IRR of 0.18 (95% CI: 0.21–0.44; *p* < 0.001), which corresponds to an 82.2% reduction; of the 32 hospitalised infants, only 17 had received nirsevimab.

Among the 17 infants hospitalised during the 2024–25 season who had received nirsevimab, one was extremely preterm (gestational age [GA]: 26 + 4 weeks) and two were moderate-to-late preterm (32 + 4 and 34 + 6 weeks). None of the immunised preterm infants required oxygen therapy or intensive care. In the same group of 17 hospitalised and immunised infants, two had a comorbidity (one with primary immunodeficiency and one with cardiopathy).

We also analysed monthly hospitalisation counts for RSV-related admissions across four epidemic seasons (November to March, 2021–22 through 2024–25).

Using a negative binomial regression model adjusted for calendar month to account for seasonality, the intervention period was associated with a statistically significant decrease in admissions (Incidence Rate Ratio [IRR] 0.25, 95% CI 0.08–0.75; *p* = 0.013). This corresponds to an estimated 75% reduction in hospitalisations during the season in which nirsevimab was used.

### RSV-related hospitalisations in infants born during the RSV season.

Among infants born during the RSV season, hospitalisations dropped from a baseline mean of 65.7 cases (71, 61, 65) in the three previous seasons to 11 cases in 2024–25, IRR 0.17 (95% CI: 0.05–0.18; *p* < 0.001), corresponding to an 83.2% reduction; 9 of these 11 infants had received nirsevimab.

Among these infants, 5/9 were immunised on the second day of life, 3/9 on the third day, and 1/9 on the fourth day. The time interval between the day of immunisation and the day of hospitalisation.

ranged from a minimum of 21 days to a maximum of 141 days (mean 51.2 days; median 45 days; IQR 30–56).

### RSV-related hospitalisations in infants born before the RSV season

In the group of infants born before the RSV season, hospitalisations declined from a baseline mean of 113.3 cases in the three previous seasons to 21 cases in 2024–25, IRR 0.19 (95% CI: 0.13–0.30; *p* < 0.001), corresponding to an 81.5% reduction; 8/21 had received nirsevimab. The time interval between immunisation and hospitalisation varied from a minimum of 53 days to a maximum of 94 days (mean: 74.3 days; median: 74.5 days; IQR: 71.25–78.5).

Subdividing further the children born before the RSV season into two quarters (April-July and August-October), in the former (children born between April and July) hospitalisations declined from a baseline mean of 40.7 cases in the three previous seasons to 9 cases in 2024–25, IRR 0.22 (95% CI: 0.12–0.42; *p* < 0.001), corresponding to a 77.9% reduction; 3/9 had received nirsevimab.

In the subgroup of infants born between August and October, hospitalisations declined from a baseline mean of 73 cases in the three previous seasons to 12 cases in 2024–25, IRR 0.16 (95% CI: 0.09–0.29; *p* < 0.001), corresponding to an 83.6% reduction; 5/12 had received nirsevimab.

In the previous years, among children within the target age for immunisation, most RSV hospitalisations occurred among infants born before the RSV season: 55.9% (90/161) in 2021–22, 69.2% (137/198) in 2022–23, 63.7% (114/179) in 2023–24.

**Fig. 1:**
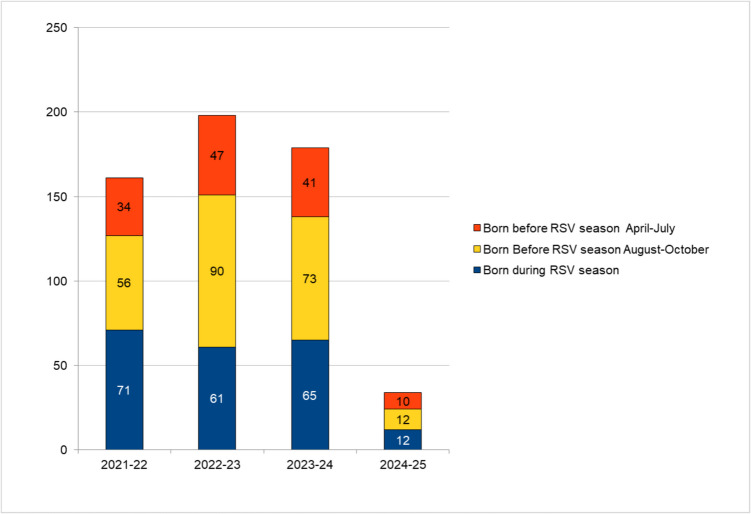
RSV-related hospitalisations in 3 seasons before the introduction of nirsevimab (2021–2024) and in the first nirsevimab season according to birth during the RSV season or before the RSV season

The group of infants born between April and July always represented a substantial proportion of RSV hospitalisation cases: 21.1% (34/161) in 2021–22, 23.7% (47/198) in 2022–23, 22.9% (41/179) in 2023–24 (Fig. 1).

### Need for respiratory support

A marked reduction in the need for respiratory support was observed during the 2024–2025 RSV season compared to the three preceding seasons 2021–24.

The mean number of hospitalisations requiring respiratory support with low-flow oxygen during the 2021–2024 seasons was 24.3 and 50.0 for in-season and out-of-season infants, respectively. In the 2024–25 season, these numbers dropped to 4 and 8, with IRRs of 0.16 (95% CI: 0.09–0.29; *p* < 0.001) and 0.16 (95% CI: 0.08–0.32; *p* < 0.001), corresponding to reductions of 83.6% and 84.0%. A similar trend was noted for high-flow oxygen, with mean values of 32 and 59.0 during the pre-nirsevimab seasons, declining to 3 and 6 in 2024–25, IRRs 0.09 (95% CI: 0.03–0.27; *p* < 0.001) and 0.10 (95% CI: 0.04–0.25; *p* < 0.001), corresponding to reductions of 90.6% and 89.8%, respectively. Use of Continuous Positive Airway Pressure (CPAP) or helmet ventilation also declined significantly, from a mean of 9.7 and 11.7 to 1 and 0 in the last season, IRRs 0.10 (95% CI: 0.01–0.50; *p* < 0.001) and 0.00 (95% CI: 0–0.30; *p* < 0.001), corresponding to reductions of 89.7% and 100%. Endotracheal intubations averaged 2.7 (in-season) and 4.3 (out-of-season) over the previous seasons. Crucially, no endotracheal intubations were required among in-season or out-of-season infants during the 2024–25 season, highlighting a notable clinical impact; the 95% confidence intervals were 0–0.37 and 0–0.38, respectively (*p* < 0.001).

### PICU Admissions

Overall, among those eligible for nirsevimab, PICU admissions fell from 54 (61, 49, 52) to 8 cases, IRR 0.15 (95% CI: 0.07–0.30; *p* < 0.001), corresponding to an 85.2% reduction. Among infants born during RSV season, PICU admissions fell from an average of 31 (37, 24, 33) to 5 cases, IRR 0.16 (95% CI: 0.06–0.38; *p* < 0.001), corresponding to an 84.0% reduction. In infants born before RSV season, the IRR was 0.13 (95% CI: 0.03–0.37; *p* < 0.001), corresponding to an 86.8% reduction (from 23 to 3 cases).

## Discussion

Analysis of our data reveals that all infants in their first RSV season need protection against RSV.

Targeting all infants born between 1 April 2024 and 31 March 2025, Tuscany’s RSV immunisation programme achieved an overall 82.1% reduction in hospitalisations among eligible infants, comparable to observations reported in other countries [15–17]. The programme demonstrated comparably high impact across subgroups, with reductions of 83.2% in infants born during the season, 81.5% in those born before the season, 83.6% in August–October births, and 77.9% in April–July births. It is especially noteworthy that infants born before the RSV season contributed to the majority of RSV hospitalisations (55.9% in 2021–22, 69.2% in 2022–23, 63.7% in 2023–24) in the pre-Nirsevimab seasons. This observation was not entirely unexpected, as comparable results.

have been documented in studies conducted in other countries, including the USA and Spain [18, 19]. We may hypothesise that infants born between April and October, although slightly older than those born during the RSV season, may have an increased risk of encountering the virus as they are exposed to the entire RSV season.

Although our study evaluates a population limited to a single centre, we believe our data are informative for other Italian regions and other countries that are considering this strategy. The resolutions of the Italian regions were patchy, so that, as an example, some regions offered nirsevimab only to infants born from August 1, 2024 to March 31, 2025, and one region offered nirsevimab only to infants born during the RSV season. As for Tuscany Region, based on surveillance data obtained in the region over the years, evaluating the distribution of hospitalisations.

among different age groups, the decision was to offer prophylaxis to all infants in their first RSV season (born from April 1, 2024 to March 31, 2025) and children up to 24 months of age who remain vulnerable to severe RSV disease through their second RSV season [2].

The most severe presentations, such as PICU admissions and the necessity for invasive or non-invasive respiratory support, demonstrated a substantial decline during the 2024–2025 RSV season. Our study observed an over 85% reduction in PICU admissions, aligning with findings by Bermúdez-Barrezueta et al. who reported an 87.9% decrease in PICU admissions following universal nirsevimab administration [20]. Similarly, the use of non-invasive CPAP decreased by a factor of 20, while high- and low-flow oxygen therapies saw reductions ranging from five to tenfold. Notably, no cases of endotracheal intubation were recorded during this period, in contrast to previous seasons. These outcomes are consistent with the study by García-García et al. which highlighted a significant reduction in oxygen therapy utilization among infants receiving nirsevimab [21].

While a formal cost–benefit analysis of universal nirsevimab immunisation is beyond the scope of this study, the comparable number of hospitalisations among infants born in different periods (April–July, August–October, November–March) suggests that a universal strategy would substantially reduce hospitalisations and the use of respiratory support therapies, thereby lowering healthcare costs and providing a significant public health benefit. These findings are supported by modelling studies from our group [12] and others [15].

This study has several limitations. It is a single-centre study with a relatively modest sample size, meaning the findings primarily reflect a specific geographic area and may not be generalisable to other settings. The low number of hospitalisations following the introduction of nirsevimab, although indicative of its impact, limits detailed analyses of subgroups. Nevertheless, our results are consistent with reports from other regions, including Luxemburg or Spain [16, 17], where similar significant reductions in RSV-related hospitalisations were observed.

## Conclusion

Overall, our study shows that a universal RSV prevention strategy with nirsevimab has a significant public health impact in terms of reducing RSV-related hospitalisations. The number of hospitalisations avoided is broadly comparable between infants born during the season and those born before the season (both those born between April and July and those born between August and October). This suggests that prioritising one subgroup over another may not be appropriate, as it would only partially alleviate the burden of RSV in infants.

## Data Availability

The datasets generated and analyzed during the current study are available from the corresponding author on request.
